# Perifollicular Melanocyte Regeneration in Bullous Pemphigoid

**DOI:** 10.7759/cureus.61652

**Published:** 2024-06-04

**Authors:** Isha Jhingan, Nicholas W Oldham, Daniel A Hyman, Jeffrey D McBride, Jarad I Levin

**Affiliations:** 1 Department of Dermatology, University of Oklahoma Health Sciences Center, Oklahoma City, USA

**Keywords:** bullous skin disease, bullous pemphigoid, melanocyte, skin of color, clinical dermatology

## Abstract

Bullous pemphigoid (BP) is an autoimmune skin disorder that causes fluid-filled blisters to appear on various body parts, often preceded by urticaria and pruritis. This case report describes the perifollicular melanocyte regeneration within diseased areas in a skin of color patient with BP. By reviewing the various pathologies that can result in melanocyte destruction and the basic science of melanocyte regeneration, we can better identify and explain this phenomenon to patients and lead to earlier diagnoses. Furthermore, due to the lack of published information on skin conditions in skin of color patients, this report can assist in raising awareness of an atypical BP presentation in the dermatological community.

## Introduction

Bullous pemphigoid (BP) often presents with tense bullae. In many cases, these blisters are preceded by pruritus and urticaria. This disease is more prevalent among elderly patients. The pathophysiology involves an immune system attack on the basement membrane zone [1]. BP180 and BP230 are the most common hemidesmosomes against which antibodies are formed [1]. These antibodies lead to inflammation through complement activation and neutrophil recruitment, ultimately destroying intercellular junctions in the dermal-epidermal layers [1]. Currently, it is unclear what triggers BP; however, antibiotics, nonsteroidal anti-inflammatory drugs, and immune checkpoint inhibitors have been reported to precede the onset of BP in some patients [2].

BP is diagnosed on both histology and immunofluorescence. Findings of bullous lesions typically include eosinophilic and neutrophilic invasion [2]. Immunofluorescence and enzyme-linked immunosorbent assays are also performed based on BP180 and BP230 proteins to support the diagnosis further [2]. Common therapies for BP include topical and systemic corticosteroids and doxycycline to mitigate the immune response and control blister formation. Corticosteroids are typically the first-line treatment to suppress the immune system and reduce the development of new lesions [1]. Doxycycline also fosters an anti-inflammatory environment by decreasing neutrophil recruitment [1]. New developments in biological therapy have also shown potential in treating BP; however, the risk of adverse events and side effects is comparatively higher with these treatments, therefore lowering their usage in clinical practice [1].

## Case presentation

**Figure 1:**
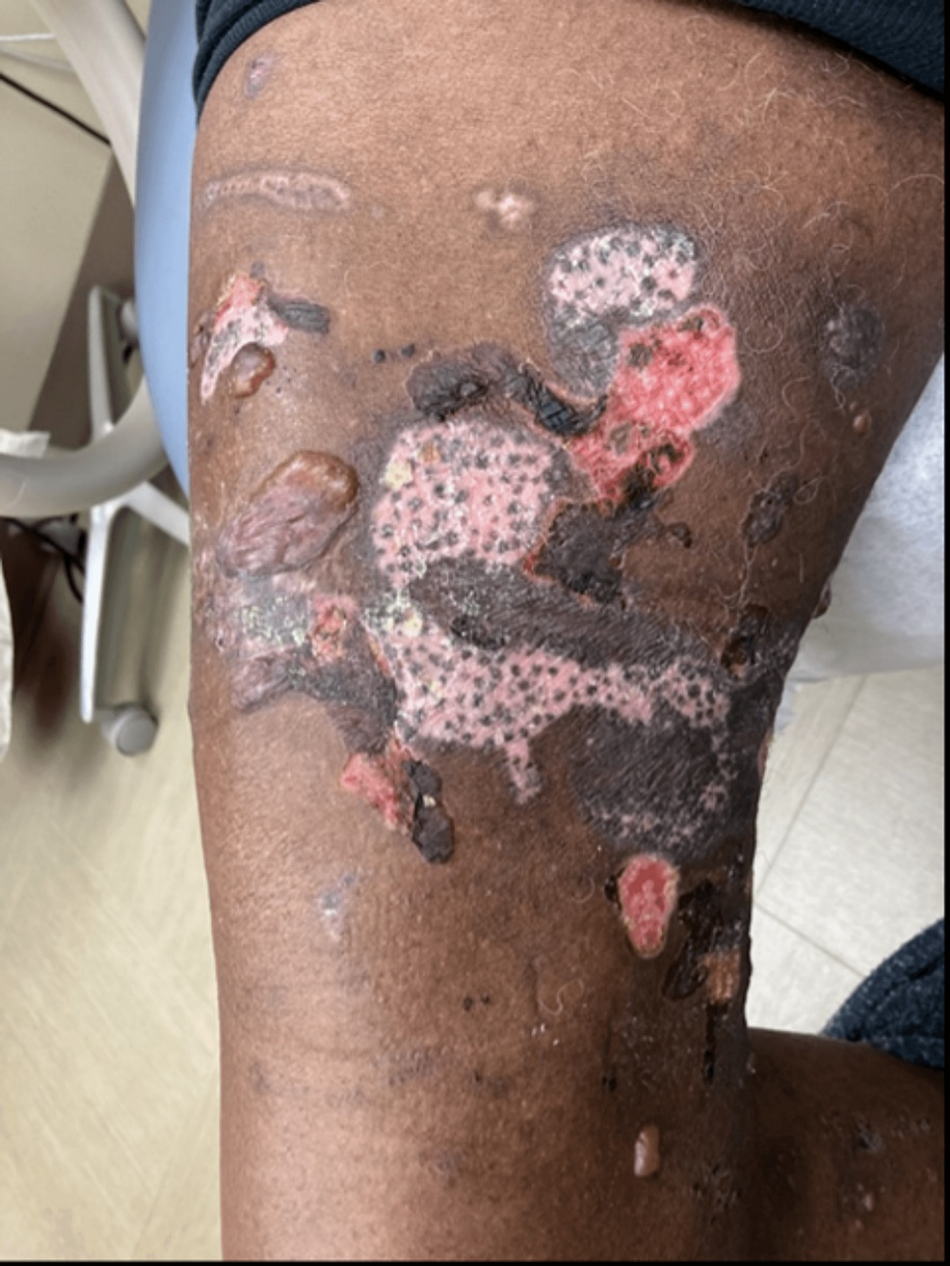
Tense bullae and ulcerated plaques with perifollicular repigmentation on the patient's right thigh

A 78-year-old African American man with no significant history of skin disease presented to the dermatology clinic with a chief complaint of itchy blisters on his trunk and extremities, which had been ongoing for several weeks. Physical examination was significant for multiple tense bullae and superficially ulcerated plaques. Striking perifollicular repigmentation was noted within ulcerated plaques on the right thigh (Figure 1). Punch biopsies for hematoxylin and eosin staining and direct immunofluorescence (DIF) analysis were performed, and the diagnosis of BP was confirmed.

**Figure 2:**
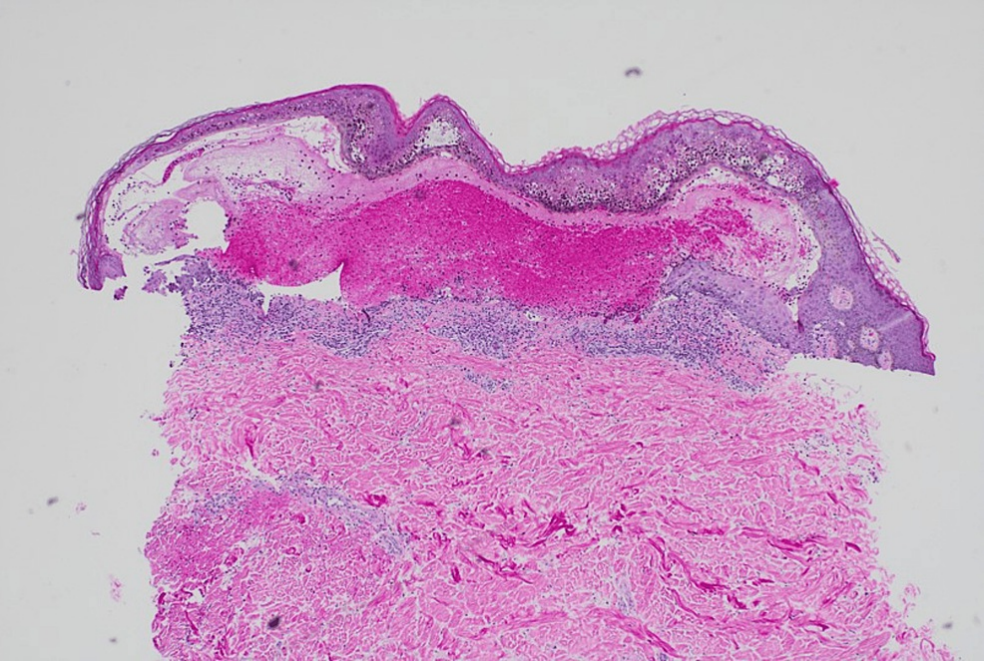
Subepidermal bulla in this case of BP (hematoxylin and eosin stain 100×) BP: bullous pemphigoid

**Figure 3:**
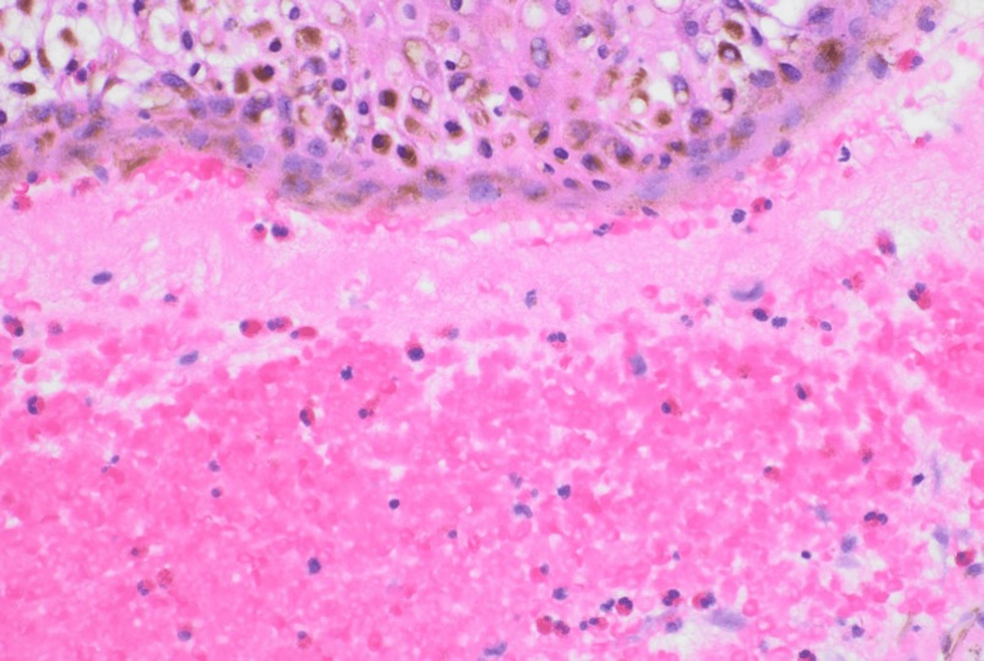
Subepidermal bulla with melanin-rich keratinocytes and subepidermal eosinophils (hematoxylin and eosin stain 400×)

Pathology demonstrated a subepidermal bulla with many eosinophils (Figures 2, 3). DIF showed basement membrane C3 deposits in a linear pattern. These results were consistent with a diagnosis of BP. Empiric treatment was started at the first visit, including one dose of intramuscular triamcinolone 40 mg, oral doxycycline 100 mg twice daily for four weeks, and triamcinolone 0.1% ointment applied to lesions as needed for itch. The patient displayed significant improvement at a two-week follow-up visit.

## Discussion

BP was diagnosed in a Fitzpatrick skin type VI patient in this case. The patient presented with a unique “polka-dot” pigment pattern, which indicated melanocyte regeneration within ulcerated plaques of affected skin. Melanocyte regeneration after destruction occurs in the bulge region of hair follicles, where melanocyte stem cells are located [3]. These melanocytes then migrate to the interfollicular epidermis and begin to multiply, with perifollicular regeneration being the typical pattern observed [3]. Multiple pathologies can destroy melanocytes and result in perifollicular repigmentation, including vitiligo or, in this case, the presentation of BP. Many dermatologists have appreciated the perifollicular regeneration of melanocytes in vitiligo patients who are recovering pigment in a diseased area of skin [3]. By reviewing the various conditions that can result in melanocyte destruction and the process of melanocyte regeneration, dermatologists can better identify and explain this phenomenon to patients.

Additionally, this case report enables a review of the presentation of a bullous skin condition in a darker skin tone. There is an increasing awareness of the lack of published and presented photographs of common diseases in the skin of color, which may result in delayed diagnosis and treatment and, ultimately, increased morbidity and mortality in this patient population [4]. For example, many patients with BP may initially present with erythematous urticarial papules and plaques that may be more difficult to appreciate in skin of color. The hair follicles regenerate as papules in BP, presenting as diffuse follicular pigmentation, as seen in this patient [5]. Given the visual nature of dermatology, it is imperative to assess the different presentations of BP in darker skin tones, as found in this case, especially when melanocyte regeneration may be involved, which may further occlude the typical presentation of this skin condition. BP is also a relatively rare skin condition; therefore, publishing cases of this condition in darker skin tones is even more imperative to allow for a quicker diagnosis and better patient outcomes.

## Conclusions

In conclusion, BP is a skin condition that could be easily misinterpreted when melanocyte regeneration is present in patients with darker skin tones due to limited published information in relation to the skin of color patients. Further research of skin conditions like BP in the skin of color patients is recommended to provide a more accurate diagnosis for a diverse patient population. While melanocyte regeneration is present in other conditions, this case presentation allows for appreciation of this phenomenon in a BP patient with darker skin tone and to evaluate a more atypical presentation of the disease.

## References

[1] Miyamoto D, Santi CG, Aoki V, Maruta CW (2019). Bullous pemphigoid. An Bras Dermatol.

[2] Moro F, Fania L, Sinagra JL, Salemme A, Di Zenzo G (2020). Bullous pemphigoid: trigger and predisposing factors. Biomolecules.

[3] Birlea SA, Goldstein NB, Norris DA (2017). Repigmentation through melanocyte regeneration in vitiligo. Dermatol Clin.

[4] Massie JP, Cho DY, Kneib CJ (2019). Patient representation in medical literature: are we appropriately depicting diversity?. Plast Reconstr Surg Glob Open.

[5] Kumaresan M, Srinivas CR (2011). Perifollicular pigmentation in bullous pemphigoid: a diagnostic sign. Indian Dermatol Online J.

